# Measuring flexibility in autistic adults: Exploring the factor structure of the flexibility scale self report

**DOI:** 10.1002/aur.3025

**Published:** 2023-09-12

**Authors:** Matthew J. Hollocks, Goldie A. McQuaid, Benjamin E. Yerys, John F. Strang, Laura G. Anthony, Lauren Kenworthy, Nancy R. Lee, Gregory L. Wallace

**Affiliations:** 1 Department of Child & Adolescent Psychiatry, King’s College London, London, UK; 2 Department of Psychology, George Mason University, Fairfax, Virginia, USA; 3 Children’s Hospital of Philadelphia, Philadelphia, Pennsylvania, USA; 4 University of Pennsylvania, Philadelphia, Pennsylvania, USA; 5 Gender and Autism Program, Children’s National Hospital, Rockville, Maryland, USA; 6 Department of Psychiatry and Behavioral Sciences, University of Colorado School of Medicine, Aurora, Colorado, USA; 7 Departments of Pediatrics, Psychiatry, and Behavioral Sciences, George Washington University School of Medicine, Washington, District of Columbia, USA; 8 Division of Neuropsychology, Children’s National Hospital, Washington, District of Columbia, USA; 9 Department of Psychological and Brain Sciences, Drexel University, Philadelphia, Pennsylvania, USA; 10 Department of Speech, Language, and Hearing Sciences, The George Washington University, Washington, District of Columbia, USA

**Keywords:** adults, autism, executive functions, factor analysis, measurement, neuropsychology, questionnaire

## Abstract

Cognitive flexibility differences are common for autistic individuals and have an impact on a range of clinical outcomes. However, there is currently a lack of well validated measurement tools to assess flexibility in adulthood. The Flexibility Scale was originally designed as a parent-report measure of real-world flexibility challenges in youth. The original Flexibility Scale provides a total score and five subscales: Routines and Rituals, Transitions and Change, Special Interests, Social Flexibility, and Generativity. In this study, we evaluate the factorial validity of the Flexibility Scale as a self-report (Flexibility Scale Self Report) measure of cognitive flexibility, adapted from the original Flexibility Scale, for use by autistic adults. This study includes both a primary sample (*n* = 813; mean age = 40.3; 59% female) and an independently recruited replication sample (*n* = 120; mean age = 32.8; 74% female) of individuals who completed the Flexibility Scale Self Report. The analysis consisted of an initial confirmatory factor analysis (CFA) of the original Flexibility Scale structure, followed by exploratory factor analysis (EFA) and factor optimization within a structural equation modeling framework to identify the optimal structure for the questionnaire in adults. The identified structure was then replicated through CFA in the replication sample. Our results indicate an alternative optimal scale structure from the original Flexibility Scale, which includes fewer items, and only three (Routines/Rituals, Transitions and Change, Special Interests) of the five subscales contributing to the flexibility total score. Comparisons revealed no structural differences within the scale based on sex assigned at birth. Here the Generativity and Social Flexibility scales are treated as independent but related scales. The implications for measurement of cognitive flexibility in clinical and research settings, as well as theoretical underpinnings are discussed.

## INTRODUCTION

Cognitive flexibility is a core domain of executive functioning (Miyake & Friedman, 2012) which encapsulates an individual’s readiness to selectively switch between cognitive processes to generate a context appropriate behavioral response (Uddin, 2021) and incorporates a range of skills including attentional and set shifting (the ability to switch back-and-forth between mental sets; Dajani & Uddin, 2015), generativity (the ability to generate spontaneously appropriate novel responses) and reward sensitivity (Hauser et al., 2015). Differences in cognitive flexibility are a common experience for autistic people and have been associated with a range of difficulties including reduced adaptive skills (Bertollo et al., 2020) and greater emotional and behavioral challenges (Hollocks et al., 2021; Lawson et al., 2015; Ozsivadjian et al., 2021). Lower levels of cognitive flexibility have also been suggested to mediate the impact of exposure to stressful life events on mental health (Carter Leno et al., 2022). To date, much of the research on cognitive flexibility in autism has focused on children and adolescents or relied on the use of performance-based measurement of cognitive flexibility (i.e., using neuropsychological tasks such as the Wisconsin Card Sort Task or similar; Dichter et al., 2010; Landry & Al-Taie, 2016; Yasuda et al., 2014).

Research into cognitive flexibility in autistic adults is hindered by the lack of a psychometrically robust and ecologically valid measure of this domain. Whilst it is recognized that autistic individuals frequently experience lower levels of cognitive flexibility, also referred to as “rigidity” or “all or nothing thinking” (Stark et al., 2021), this is not consistently found in the limited research using performance-based measures (Di Sarro et al., 2022; Landry & Al-Taie, 2016). It is probable that performance on these tasks is contaminated by difficulties across related domains, such as intellectual and/or verbal ability and broader executive functioning differences (Demetriou et al., 2018). Due to the inconsistency between the experience of cognitive flexibility differences and findings using performance-based measures, the importance of access to an ecologically valid measure of cognitive flexibility is paramount and has previously been highlighted in the literature (Geurts et al., 2009). This is vital, not only as a tool to identify flexibility challenges, but also ways in which reduced flexibility may be a strength, one that could be fostered in collaboration with autistic people.

The Flexibility Scale is a questionnaire originally designed as a parent-report measure of real-world flexibility challenges in youth (Strang et al., 2017). The development of the Flexibility Scale and item selection were based on several established components of flexibility, which are known characteristics for some autistic individuals. These include routinized thinking, passionate interests, insistence on sameness, rigidity, and generativity challenges. Importantly, the Flexibility Scale is intended to complement existing measures of restricted and repetitive behaviors or behavioral flexibility, by focusing on the everyday expression of an inflexible or flexible thinking style. In autistic youth, the Flexibility Scale was determined to have five constituent factors: Routines and Rituals, Transitions and Change, Special Interests, Social Flexibility, and Generativity. The Flexibility Scale has shown both good convergence with performance-based measures of cognitive flexibility and the ability to distinguish youth with and without an autism diagnosis (Strang et al., 2017).

Despite the increasing interest and research into autism in adulthood, self-reported flexibility challenges have rarely been investigated, and the limited evidence to date has been derived from studies utilizing broader self-report measures of everyday executive functioning, including the Behavior Rating Inventory of Executive Function-Adult version (Davids et al., 2016; Geurts et al., (2020). The use of the Flexibility Scale in adult samples has yet to be evaluated. Given the potential impact of cognitive flexibility differences for autistic adults, across domains such as adaptive skills and co-occurring psychiatric symptoms (Wallace et al., 2016), it is vital that researchers and clinicians have an effective measure of this construct validated for this age group. A validated cognitive flexibility measure for adults is also relevant for scientists studying cognitive flexibility differences in middle and late adulthood because this skill may decline with age (Berry et al., 2016). Therefore, this study aims to investigate the factor structure of the Flexibility Scale Self Report in a large sample of autistic adults recruited from the Simons Powering Autism Research (SPARK; The SPARK Consortium, 2018) participant registry. The primary aim is to evaluate the original factor structure based on youth in an adult sample using a self-report version of this tool. If we determine the factor structure from youth is inadequate for a self-report measure in adults, then we will explore alternative factor structures. To ensure our initial findings are robust, we will replicate the final factor structure in an independent sample of autistic adults.

## METHODS

### Participants

#### Primary study sample

Autistic adults were recruited through the Research Match service of the Simons Powering Autism Research (SPARK; The SPARK Consortium, 2018) participant registry (Project Numbers: RM0045Wallace1839 and RM0045Wallace4090). SPARK, part of the Simons Foundation Autism Research Initiative, maintains an active and growing registry of more than 100,000 autistic persons residing in the United States. More information about SPARK can be found here: https://sparkforautism.org/.

Participants completed an online battery of questionnaires, including the Flexibility Scale Self Report, as part of a broader study of autistic adult outcomes. Data were collected during December 2019 and January 2020. Inclusion criteria for the current study included aged ≥18 years and no diagnosis of co-occurring intellectual disability. As an additional exclusionary criterion for the current study, any participants missing ≥20% of items on the Flexibility Scale Self Report were excluded from analyses. The final sample of SPARK participants consisted of 813 autistic adults (59.0% designated female at birth; 9.96% gender diverse) aged 18.2–83.3 years.

SPARK participants were “independent adults,” defined by SPARK as persons ≥18 years of age who do not have a court-appointed legal guardian and therefore provide consent for themselves. Based on SPARK’s determination of “independent adult,” these participants are unlikely to have a co-occurring intellectual disability. Further, as part of a detailed medical history collected in the current study, no participant reported a diagnosis of an intellectual disability.

Of 813 participants, 810 (99.6%) self-disclosed a community-based professional diagnosis of an autism spectrum condition. Although SPARK does not independently confirm diagnoses, SPARK partners with and recruits from expert autism clinical sites, in part, to increase the likelihood that participants have a professional autism diagnosis (The SPARK Consortium, 2018). A separate validation study that examined electronic medical records for 254 SPARK participants, including “independent” adults, confirmed an autism spectrum diagnosis in 98.8% of the sample (Fombonne et al., 2022). Additionally, to characterize the current sample, autistic traits were queried using the 28-item Autism-Spectrum Quotient (AQ-28; Hoekstra et al., 2011), and consistent with the self-disclosed community-based autism diagnosis by nearly all of the sample, 94.83% of participants scored above the AQ-28 cut-off (>65) (Table 1).

#### Replication study sample

An independent group of autistic adults was recruited as part of a broader online study to learn about compensatory strategies utilized in everyday life by autistic adults. Participants completed a questionnaire battery, which included the Flexibility Scale Self Report. This study pursued two primary approaches for recruitment. First, recruitment included ResearchMatch, a national health volunteer registry created by several academic institutions and funded by the United States National Institutes of Health through a Clinical Translational Science Award program. ResearchMatch is an online recruitment platform connecting registered researchers with volunteers who are interested in receiving information about participation in health-related studies. In addition to ResearchMatch, study advertisements were posted to select online platforms, including public and private autism-related Facebook groups (with prior approval of the relevant group’s moderator), through the lab website of one of the authors (G.L.W.), and through Autism Science Foundation’s directory of autism research studies. Exclusionary criteria for the broader study were ≤18 years of age, self-disclosed diagnosis of an intellectual disability, or current residency outside of the United States. Participants were excluded from the current study if ≥20% of items on the Flexibility Scale Self Report were missing. Data reported on here were collected between January 2022 and September 2022.

For the purposes of the current study, the final replication sample consisted of 120 autistic adults (74.2% designated female at birth; 28.3% gender diverse) aged to 18–80 years. Of the 120 participants, 105 reported a professional community-based diagnosis of an autism spectrum condition. To characterize autistic traits in the sample, participants completed the AQ-28, and 96.67% of the sample scored above the AQ-28 cutoff (>65).

Both studies were approved by The George Washington University Institutional Review Board and followed all procedures in accordance with the Declaration of Helsinki.

### Measures

#### Autism Quotient—28

The Autism Quotient—short form (Hoekstra et al., 2011) is a 28-item self-report questionnaire where participants are asked to read descriptive statements assessing presence of autistic traits and rate these on a 4-point Likert scale, with answer categories “1 = definitely agree”; “2 = slightly agree”; “3 = slightly disagree” and “4 = definitely disagree.” The AQ-28 has been shown to have good sensitivity and specificity to detect autism, with scores of ≥65 being indicative of an autism diagnosis, and in this study is used descriptively to quantify autistic traits. For an overview of psychometric properties of the AQ-28 see Hoekstra et al. (2011).

#### The Flexibility Scale self report (Strang et al., 2017)

**FIGURE 1:**
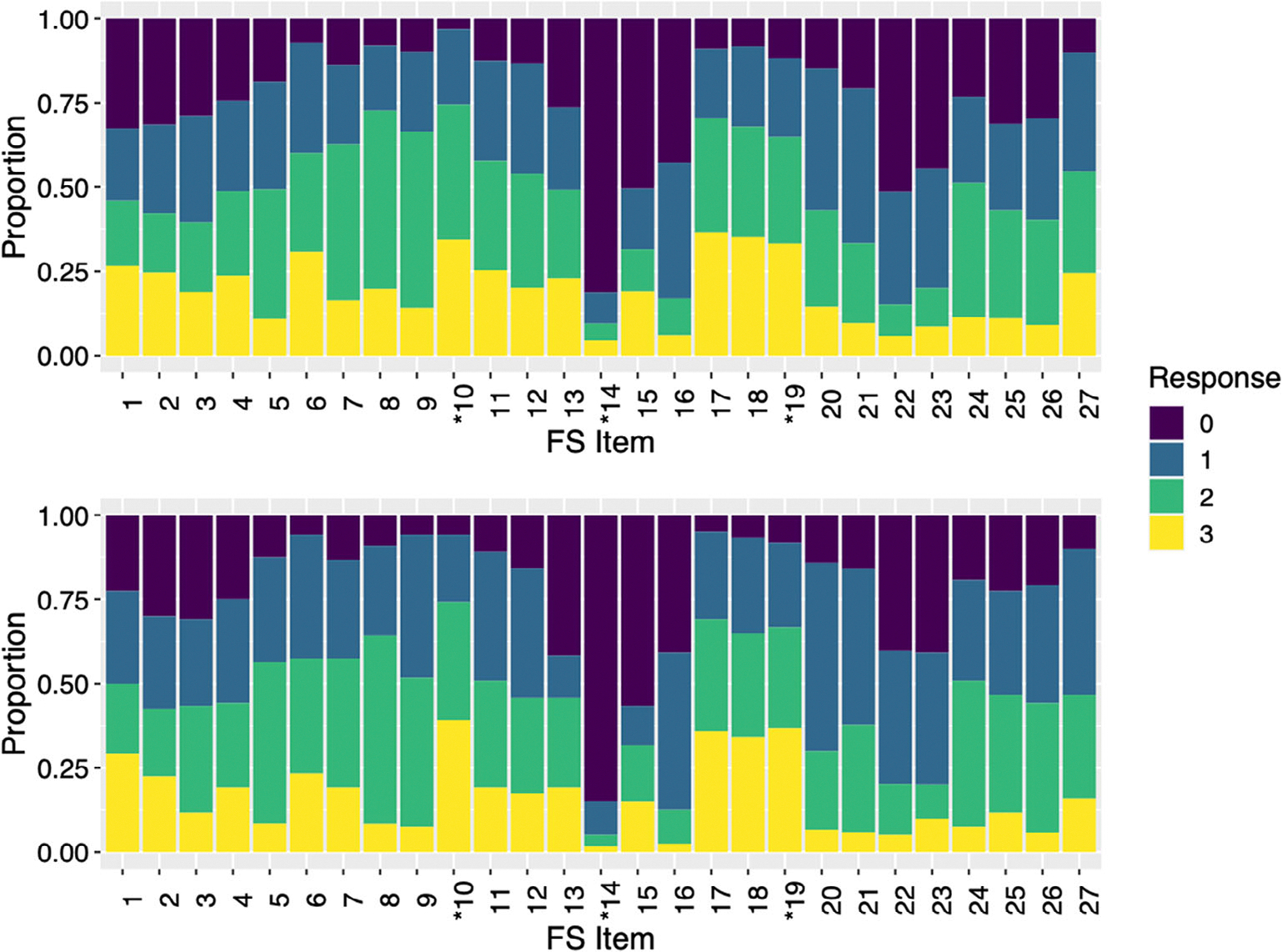
Proportion of FS item responses in the Primary (a) and Replication study (b) samples. Asterisks indicate items that are part of the original FS but were removed from the adult self-report FS. Responses: 0 = No or not true; 1 = Somewhat or sometimes true; 2 = Very much or often true, 3 = Almost always or always true. *Indicates items that were dropped from the scale prior to PCA.

In this study, the Flexibility Scale was administered to adults as a self-report questionnaire, with the purpose to evaluate the factor structure of the scale when used in this way. Item wording was adapted from the original Flexibility Scale by adapting the verbs of each item and removing any child specific language (see Table 2; Figure 1 for breakdown of item endorsement rates). The adapted Flexibility Scale Self Report consists of 27 items, responded to on a four-point ordinal Likert scale for each item: “0 = No or not true,” “1 = Somewhat or sometimes true,” “2 = Very much or often true much,” “3 = Almost or always true.” Higher scores (after reverse scoring of 9 items) mean greater endorsements of flexibility challenges. Based on initial factor analytic work using the parent/caregiver report version of the Flexibility Scale, items load onto five subscales, namely (1) Routines and Rituals, (2) Transitions/Change, (3) Special Interests, (4) Social Flexibility, and (5) Generativity (Strang et al., 2017). Subscales had high internal consistency: Routines and Rituals (*α* = 0.750), Transitions/Change (*α* = 0.906), Special Interests (*α* = 0.795), Social Flexibility (*α* = 0.854), and Generativity (*α* = 0.878) and good convergent and divergent validity. For an overview of psychometric properties of the original Flexibility Scale AQ-28 see Strang et al. (2017).

#### Statistical analysis

**FIGURE 2:**
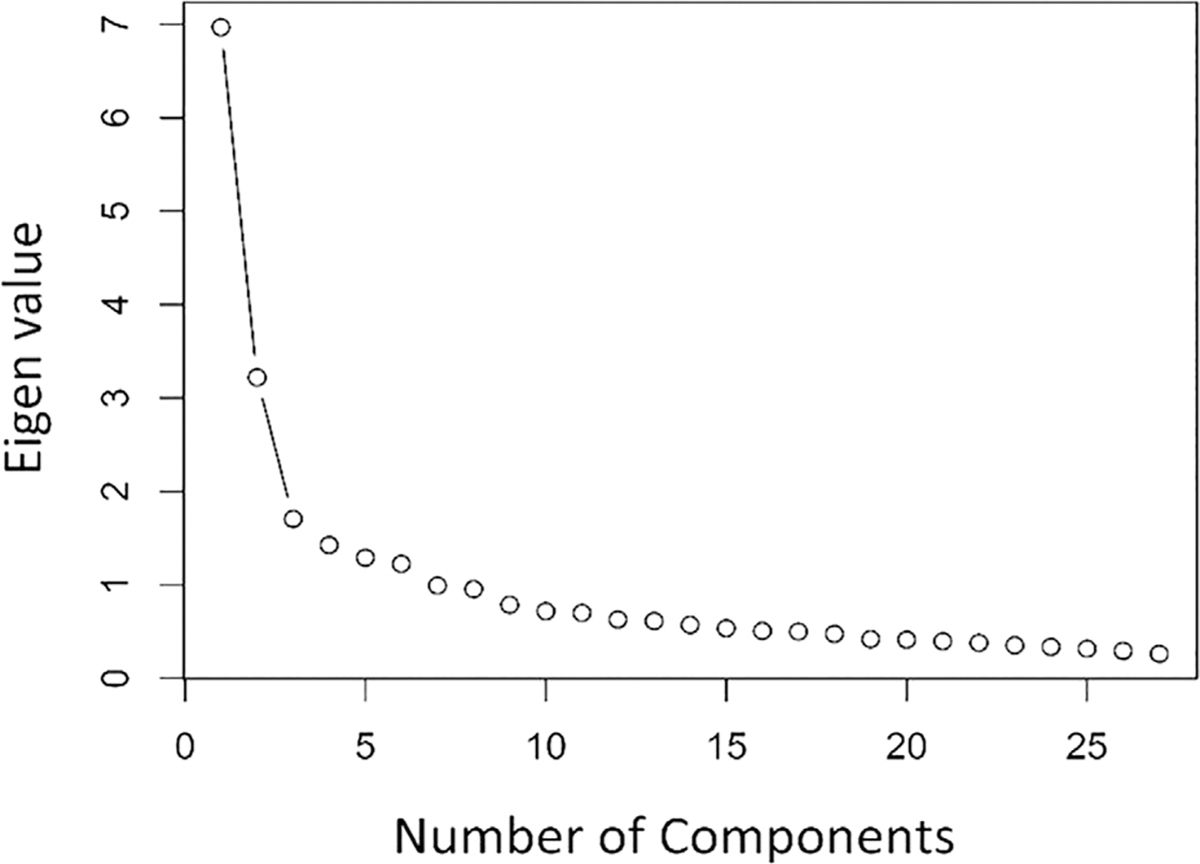
Scree plot showing inflection points for number of principal components in the primary sample.

First, the basic psychometrics of the existing five-factor scale were investigated in the Flexibility Scale Self Report with the primary sample, including establishing the internal consistency of each subscale via Cronbach’s alpha and item-item and item-total correlations. Next, an initial confirmatory factor analysis (CFA) was completed to test the suitability of the original factor structure. In line with the use of an oblique rotation in the original exploratory factor analysis (EFA), subscales were allowed to correlate, and a diagonally weighted least squares (DWLS) estimator was used to account for ordinal item level data (Li, 2016). Model-fit was evaluated using the comparative fit index (CFI) and root mean square error of approximation (RMSEA). A good model fit is indicated by the above fit statistics including a chi-square likelihood ratio test *p*-value ≥ 0.05, CFI ≥ 0.95 and a RMSEA ≤ 0.08 (Hu & Bentler, 1999). Based on these initial results a principal components analysis (PCA) with *oblimin* rotation was conducted on the sample to identify if an alternative factor structure better explained the observed data. Oblimin rotation was selected to allow components to correlate (Osborne, 2015), which is consistent with both theoretical considerations (that the factors of the scale represent associated constructs) and the initial development of the Flexibility Scale. Bartlett’s test and Kaiser-Meyer-Olkin (KMO) were used to ensure items were adequately correlated and that PCA is a suitable approach for the data. A scree plot was generated (see Figure 2), and eigenvalues examined to determine how many components should be included. For EFA, we applied a minimum valid factor loading of 0.38, with those items not meeting this threshold being removed. Whilst there are few accepted norms for factor loading cut-offs, a minimum loading between 0.30 and 0.40 (Tavakol & Wetzel, 2020) is generally recommended, with the current, more conservative cutoff selected to reduce cross-loading and to be consistent with previous work (Magiati et al., 2017). In the case of cross-loading items which met the factor loading requirements, these were included in the factor in which they loaded most strongly only. Having completed the PCA, we conducted factor optimization within a structural equation modeling framework to identify the optimal structure for the questionnaire in adults. This consisted of comparing model-fit statistics (see above) of the baseline model with one in which nonsignificant paths were constrained to have zero variance. Finally, having established an optimal factor structure in the SPARK sample of autistic adults, a second CFA was conducted in the replication study sample of autistic adults to provide independent replication.

## RESULTS

### Descriptive statistics

Full descriptive statistics for both the primary and replication studies autistic adult samples are presented in Table 1. The primary study autistic adult sample was significantly older (primary sample mean = 40.3 years; replication study mean = 32.8 years; *p* < 0.0001); whilst the replication study autistic adult sample had a greater proportion of participants assigned female sex at birth (74.2% vs. 59%) and more participants with a bachelor’s degree or higher (63.33% vs. 44.4%). In accordance with findings of proportional over-representation of gender identity diversity among autistic populations (van der Miesen et al., 2018), 9.98% of the primary and 28.3% of the replication sample was gender diverse (i.e., individuals who experience their gender identity as different from their assigned sex at birth). Figure 1 displays the response frequency across each of the original Flexibility Scale items for both the primary and replication samples. There was a good distribution of scores across most items, apart from item 10, “I closely follow rules,” and item 14, “I often pretend to be the same character.”

### Confirmatory factor analysis of the original flexibility scale factor structure

Initial psychometrics of the original Flexibility Scale items indicated good internal consistency across subscales with Cronbach’s alpha ranging between 0.73 and 0.82. Item-total correlations ranged between *r* = 0.12–0.67; with the lowest of these being an item from the Transitions/Change subscale, “I closely follow rules,” which was considerably lower than the other items (with the next lowest being *r* = 0.21). The CFA was then completed based on the original five factor solution with items loading onto their corresponding factors; Routines and Rituals; Transitions/Change; Special Interests; Social Flexibility and Generativity. A second order flexibility total score latent variable was included onto which each of the five factors loaded. Overall, the model fit of the original Flexibility Scale factor structure was found not to be adequate (*χ*^2^(247) = 3510.07, *p* < 0.001; CFI = 0.81, RMSEA = 0.104; 90% CI = 0.100–0.107). All items strongly and significantly loaded onto their respective factors (all *p* < 0.001), except for the item “I closely follow rules,” which loaded significantly but with reduced magnitude (*B* = 0.114; *p* = 0.017). Each of the five factors loaded significantly onto the flexibility total latent variable (all *p* < 0.01). To explore the potential difference in factor structure based on sex designated at birth we re-ran the CFA separately for assigned females (*n* = 480) and assigned males (*n* = 332) revealing no difference in factor loadings or model fit. Given the size of the sample, we did not explore factor analytics by gender identity diversity in addition to assigned sex at birth. Although we did observe the expected proportional overrepresentation of gender diversity in this autistic sample, this still represented only ~10% of the sample (e.g., 81 people), which is an insufficient number of individuals with which to achieve stable factor analytics.

### Principal component analysis

Given the sub-optimal model fit of the original parent/caregiver report version Flexibility Scale factor structure to our adult self-report version, a principal component analysis (PCA) was conducted. The PCA was initially conducted with all 27-items, with oblimin rotation. The Kaiser-Meyer-Olkin test of sampling adequacy was excellent at 0.89. Bartlett’s test of sphericity was found to be significant (*p* < 0.001) indicating that the inter-item correlations were adequate for PCA. The points of inflection on the scree plot indicated that four, five or six components were optimal (Figure 1), with six components having eigen values >1. Examination of item loading for both four and six components revealed significant cross loading, and a five-factor solution was identified as the best compromise between variance explained (55%) and minimal item cross-loading. Several items had low factor loadings <0.38 and these were excluded; the item “I closely follow rules” was excluded prior to running the final five factor solution as it has consistently underperformed in preliminary analyses and was under-endorsed across the samples (see Figure 1). Two additional items were dropped due to not meeting the >0.38 threshold, these were “I often pretend to be the same character” and “I enjoy categorizing information” from the Special Interests subscale. The resulting five components, consisted of 24 items and closely resembled the original five factors of the Flexibility Scale, with the exception that the item “I insist on carrying around something with me” moved from the Routines and Rituals to the Special Interests subscale and “I build on ideas of others in conversations,” moved from the Generativity to the Social Flexibility subscale. See Table 2 for the final five factor solution and individual item loadings.

### Factor optimization of proposed scale structure within a structural equation modeling framework

**FIGURE 3:**
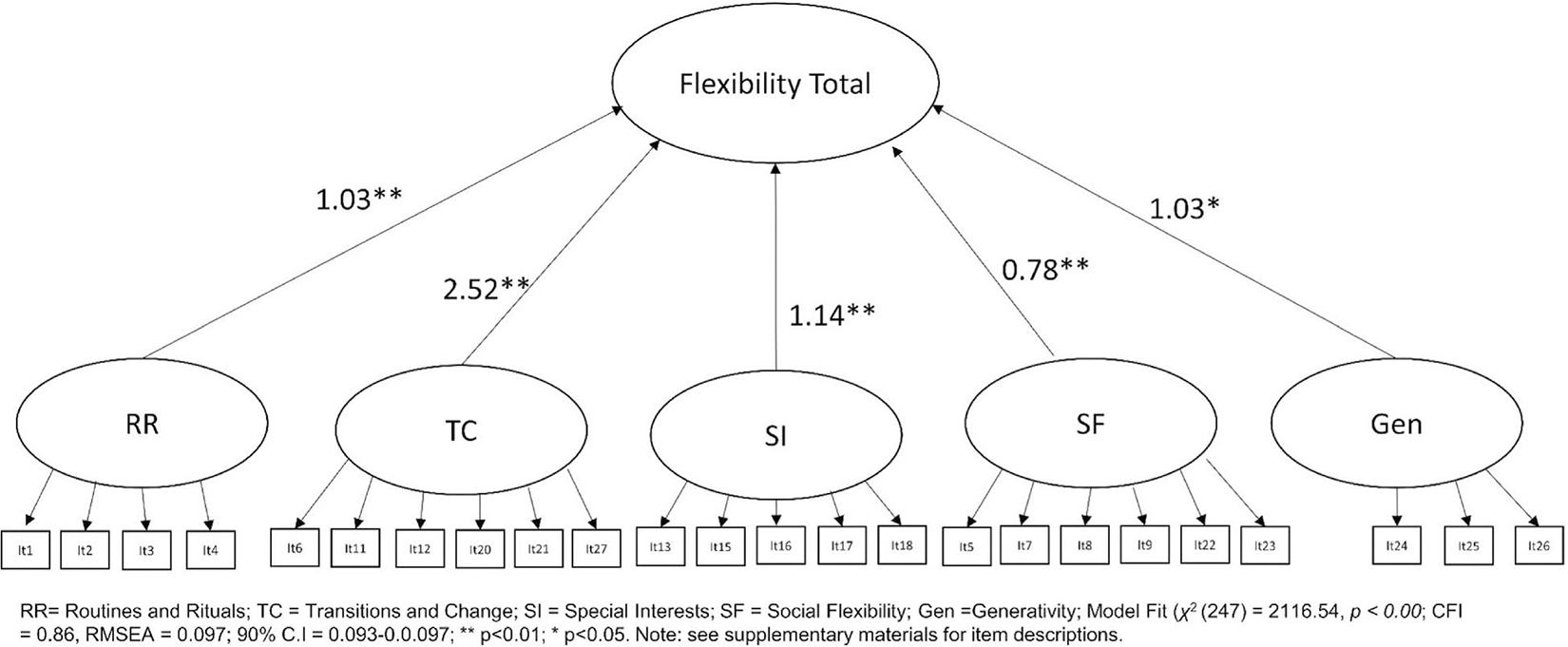
Confirmatory factor analyses for the five-factor flexibility total score in the primary sample.

**FIGURE 4:**
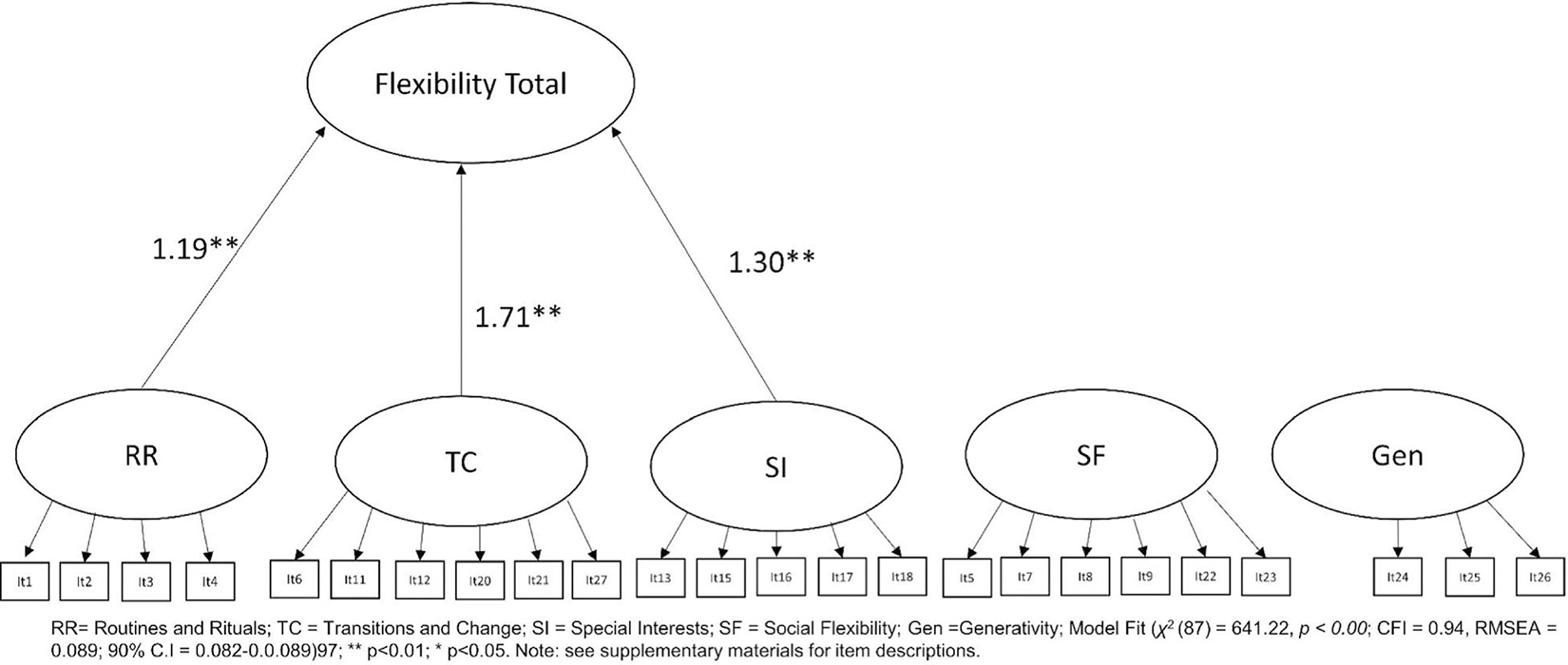
Optimal three-factor flexibility total score with Social Flexibility and Generativity as independent indices in the primary sample.

Having used PCA to identify the optimal factor structure in the primary autistic adult sample, the CFA was repeated. This indicated an improved, but still below adequate model fit (*χ*^2^(247) = 2116.54, *p* < 0.001; CFI = 0.86, RMSEA = 0.097; 90% CI = 0.093–0.0.097; see Figure 3). As expected, based on the EFA, all items loaded significantly onto their respective factors; with each factor in turn loading significantly onto a second order total flexibility latent variable: Routines and Rituals (*B* = 1.03; *p* < 0.001), Transitions/Change (*B* = 2.52; *p* < 0.001), Special Interests (*B* = 1.14; *p* < 0.001), Social Flexibility (*B* = 0.78; *p* < 0.001), and Generativity (*B* = 0.09; *p* = 0.04). It was noted that whilst each of the subscales significantly loaded onto the flexibility total latent variable, the Social Flexibility and Generativity scales loaded to a lesser extent. We then ran a simplified model in which only Routines and Rituals (*r*^2^ = 0.52); Transitions/Change (*r*^2^ = 0.86) and Special Interests (*r*^2^ = 0.56) were retained loading onto a Cognitive Flexibility Total Score. This resulted in a substantially improved model fit (*χ*^2^(87) = 641.22, *p* < 0.001; CFI = 0.94, RMSEA = 0.089; 90% CI = 0.082–0.0.089; see Figure 4), with each of these subscales continuing to significantly load onto the Cognitive Flexibility Total Score variable (all *p* < 0.001).

### Replication of factor structure in an independent sample

The final model including only the simplified three factor structure identified from the analysis on the SPARK autistic adult sample was then replicated on participants from the *replication study* autistic adult sample. Again, using robust DWLS, we found that this model had adequate fit to the data (*χ*^2^(87) = 131.35, *p* < 0.001; CFI = 0.95, RMSEA = 0.065; 90% CI = 0.041–0.0.065). As before, the Routines and Rituals (*B* = 4.5; *p* ≤ 0.001; *r*^2^ = 0.39); Transitions/Change (*B* = 1.23; *p* < 0.001; *r*^2^ = 0.91); and Special Interests (*B* = 5.28; *p* < 0.001; *r*^2^ = 0.48) subscales all loaded significantly onto the flexibility total latent variable. As with the five-factor model in the SPARK autistic adult sample, which indicated that each of the factors significantly loaded onto the Cognitive Flexibility Total score, the overall model fit of the five-factor model was inferior to the three-factor Flexibility Scale model (*χ*^2^(247) = 451.32, *p* < 0.001; CFI = 0.85, RMSEA = 0.083; 90% CI = 0.071–0.0.095).

### Subscale inter-correlations and associations with autism symptom severity

In the SPARK autistic adult sample, the three-factor Flexibility Total Score was correlated with Social Flexibility (*r* = 0.38, CI = 0.32–0.44; *p* < 0.05) but not Generativity (*r* = −0.02; CI = −0.09 to 0.05), whilst Social Flexibility and Generativity were correlated with each other (*r* = 0.28; *p* < 0.05). AQ-28 total score was significantly correlated (*p* < 0.05) with the Flexibility Total Score (*r* = 0.51), Social Flexibility (*r* = 0.45) and Generativity subscale scores (*r* = 0.17). The same pattern of results was found in the replication study autistic adult sample, with the three-factor Flexibility Total Score being correlated with Social Flexibility (*r* = 0.30; CI = 0.10–0.48, *p* < 0.05) but not Generativity (*r* = −0.02; CI = −0.27 to 0.11) with the exception being that the AQ-28 total score was not correlated with the Generativity subscale (*r* = −0.06; CI = −0.23 to 0.11).

## DISCUSSION

The aim of this study was to investigate the factor structure of the Flexibility Scale Self Report in two relatively large samples of autistic adults. The Flexibility Scale was originally designed as a parent/caregiver informant questionnaire for young people and had yet to be evaluated as a self-report measure for use by autistic adults. Having used a combination of both EFA and CFA in two independent samples of autistic adults to identify and replicate the optimal factor structure, we found that the structure of the questionnaire remained largely unchanged. This is with the exception that two items were dropped from the questionnaire, and two further items switched subscale membership. By comparing model-fit across competing factor structures we identified that the optimal structure for the questionnaire is one in which only three of the subscales (Routines and Rituals, Transitions/Change and Special Interests) are included as a part of a second order Cognitive Flexibility Total Score, whilst the remaining two subscales (Social Flexibility and Generativity) are treated as separate indices. This is the main difference between the current findings using the Flexibility Scale Self Report when compared to the initial validation study of the parent/caregiver report version of the Flexibility Scale in children and adolescents (Strang et al., 2017). These changes may represent developmental differences in the underlying constructs, or alternatively could be related to how autistic adults self-report on the everyday experiences of these cognitive processes. It is also relevant to highlight that during the original scale development, generativity was found to be less correlated with the Flexibility Scale Total Score.

As described during the original development of the Flexibility Scale, the questionnaire was designed to capture real-world flexibility challenges (Strang et al., 2017), which are encapsulated by the five subscales measuring routines and rituals; transitions and change; special interests; social flexibility; and generativity. Whilst the current findings mostly support this theoretical underpinning, we found that social flexibility and generativity loaded less strongly onto the overarching flexibility latent variable, suggesting that these constructs may be better considered separate indices of behaviors associated with, but distinct from, cognitive flexibility. This is consistent with the original validation in youth with the Generativity subscale being found to have a lower magnitude correlation with the Flexibility Scale Total Score compared to the other subscales and was not significantly correlated with the Routines and Rituals, Transitions/Change, or Special Interests’ subscales (Strang et al., 2017). It was particularly noteworthy that the Generativity subscale was not significantly correlated with the three-subscale Cognitive Flexibility Total Score. Whilst our results suggest generativity challenges could be considered distinct from difficulties with flexibility, it will likely remain a helpful index of relative strengths and weakness in this area alongside those indices assessing flexibility and social difficulties. Problems with generativity are commonly reported in autistic people (Dichter et al., 2009), and indeed they were found to be correlated with autistic traits in the SPARK autistic adult sample.

In the current study the Social Flexibility subscale was also found to load less strongly onto the overall flexibility latent construct (although Social Flexibility loaded more strongly than Generativity), and model-fit again indicated that this may benefit from being treated as an independent index. The items included in the Social Flexibility subscale primarily included those which asked about flexibility in the context of social interactions, including the ability to take turns and share possessions. One item, previously found in the Generativity subscale, “I build on ideas of others in conversation” loaded onto the Social Flexibility subscale. Despite having a clearly social emphasis, previous findings have shown the Social Flexibility subscale of the Flexibility Scale to correlate with performance measures of “switching” (Strang et al., 2017), and associations between social ability and executive functioning have been previously described in autistic youth (Kenworthy et al., 2014; Pellicano, 2007).

The finding that the Flexibility Scale Self Report is significantly associated with autistic traits across both samples is consistent both with findings using parent/caregiver reports in autistic youth (Strang et al., 2017), and the current theoretical understanding that reduced flexibility is commonly experienced by autistic people (Uddin, 2021). However, it should also be noted that evidence suggests the associations between reduced flexibility and challenges such as anxiety and behavioral difficulties, can be distinguished from associations with other symptoms such as restricted and repetitive behaviors (Hollocks et al., 2021). This is important as it may allow further specificity in understanding how heterogeneity in different features of autism may act as risks or resilience factors for different outcomes, and which of these may be modifiable factors that can be targeted by interventions. In particular, the role of flexibility as an area of strength for autistic individuals has yet to be explored.

Practically speaking, our results suggest that in future studies with autistic adults, researchers may wish to consider not using the Flexibility Scale Total Score but rather, the three-factor Cognitive Flexibility Total Score, Social Flexibility and Generativity as three separate indices. This is both in line with the optimal factor structure but may also allow further granularity when studying associations between measures of interest and behavioral reports of cognitive flexibility, social inflexibility and generativity challenges. From a theoretical perspective, it is important to consider the role of constructs such as generativity in behavioral manifestations of flexibility differences, and whether there are changes in this association throughout development.

### Limitations and future research

The current study has several strengths, including the use of two independent samples of autistic adults with good representation across multiple demographic and background variables including age, birth-sex, gender identity, and educational attainment. Whist on balance this can be considered a strength, an associated limitation is that there are high levels of heterogeneity within the samples, but with a sample size that does not allow detailed examination of the impact of this heterogeneity on the Flexibility Scale Self Report factor structure in the current study. For example, future research into measurement of cognitive flexibility in those aged 65 and above, and possible associations with aging would be warranted. Also, given the high level of gender diversity reported within this and other samples of autistic adults, future research to understand how this may lead to structural changes in the reporting of flexibility will be important. Similarly, one future direction would be to collect data in a more racially balanced sample. It is also important to note that our sample did not include autistic adults with an intellectual disability. Therefore, this scale has not been validated for use with this subgroup, and this should be an additional focus of future research. It is also relevant to note that this study only sampled from the US, and therefor findings may not be generalizable to other countries or settings.

Other areas for ongoing consideration would be the investigation of the convergent and divergent validity of the Flexibility Scale Self Report in adults, using performance-based measures of flexibility and other questionnaires designed to measure autistic traits, respectively. Indeed, as an alternative factor structure for the Flexibility Scale Self Report has been suggested for use with adults, further work on establishing its psychometric properties across the range of validity parameters, including convergent and divergent validity with additional metrics of autistic traits, and performance-based measures of executive functioning is required. This is particularly relevant given our finding that Social Flexibility and Generativity appear to be best considered as independent indices from the broader construct of cognitive flexibility. Given the implications of reduced flexibility for a range of physical and mental health outcomes in autistics adults (Hollocks et al., 2021) and that it could be a target for future interventions for autistic adults (as it has been for children; Kenworthy et al., 2014), assessments of the Flexibility Scale’s test–retest reliability and sensitivity to change are vital next steps in its adaptation for use with adults. It will also be of interest to compare findings of self-report measures of flexibility with more ecologically valid tasks-based measurement, such as the “challenge task” currently being developed for use with adult populations, but designed for youth (Kenworthy et al., 2020). Nonetheless, this investigation provides preliminary support for the utility of the first adult self-report measure of flexibility.

## Figures and Tables

**TABLE 1:** Primary and replication study samples: Participant characteristics.

	Primary sample *N* = 813	Replication sample *N* = 120	Test statistic, *p*-value, effect size

**Age, years**			
Mean (SD)	40.3 (13.9)	32.8 (12.9)	*t*(931) = 5.59, *p* < 0.0001,
Median (Range)	39 (18.2–83.3)	29 (18–80)	*d* = 0.56
**Sex assigned at birth, *n* (%)**			*χ*^2^(1) = 9.96, *p* = 0.0014,
Female	480 (59.0%)	89 (74.2%)	*V* = 0.10
Male	332 (40.8%)	31 (25.8%)	
Missing	1 (0.12%)	-	
**Gender identity, *n* (%)**			*χ*^2^(1) = 32.50, *p* < 0.0001,
Gender diverse	81 (9.96%)	34 (28.3%)	*V* = 0.19
Cisgender	730 (89.79%)	86 (71.7%)	
Not reported	2 (0.25%)	-	
**Ethno-racial identity, *n* (%)**			*χ*^2^(6) = 10.23, *p* = 0.09,
*Race*			*V* = 0.11
African American or Black	18 (2.21%)	3 (2.50%)	
Asian	13 (1.60%)	5 (4.17%)	
More than one race	82 (10.10%)	11 (9.17%)	
Native American/Native Alaskan	8 (0.98%)	1 (0.83%)	
Other	18 (2.21%)	0 (0%)	
Unknown	-	1 (0.83%)	
White	671 (82.53%)	99 (82.50%)	
Not reported	3 (0.37%)	-	
			*χ*^2^(2) = 0.92, *p* = 0.66,
*Ethnicity*			*V* = 0.03
Latinx	68 (8.36%)	7 (5.83%)	
Not Latinx	727 (89.42%)	110 (91.67%)	
Unknown	12 (1.48%)	2 (1.67%)	
Not reported	6 (0.74%)	1 (0.83%)	
**Educational attainment, *n* (%)**			*χ*^2^(1) = 9.96, *p* = 0.002,
Less than a bachelor’s degree	450 (55.35%)	44 (36.67%)	*V* = 0.10
Bachelor’s degree or higher	361 (44.40%)	76 (63.33%)	
Not reported	2 (0.25%)	-	
**AQ-28 total raw score** ^a^			*t*(930) = 0.26, *p* = 0.80,
Mean (SD)	84.6 (11.6)	84.3 (11.3)	*d* = 0.03
Median (Range)	85 (47–112)	85 (61–109)	
**AQ ≥ 65, *n* (%)**			*χ*^2^(1) = 0.67, *p* = 0.50,
Yes	771(94.83%)	116 (96.67%)	*V* = 0.03
No	41 (5.04%)	4 (3.33%)	
Missing	1 (0.13%)	-	

Abbreviation: AQ-28, 28-item Autism-Spectrum Quotient.

a SPARK sample, AQ-28 *N* = 812.

**TABLE 2:** Items and factor loadings for the 24-item Flexibility Scale Self-Report (FS-SR) and the 27-item informant-report Flexibility Scale (FS).

Item #	Item wording in FS-SR	New 24-item FS-SR factor loading	Original 27-item FS factor loading

1	I do something special around bedtime (e.g., I must fold my blanket over the pillow before I can go to sleep)	Routines and Rituals	Routines and Rituals
2	I have something special that must be done when starting my day (e.g., in the morning I must first hang up my coat, then get a drink of water, then say good morning to my instructor/friend/coworker)	Routines and Rituals	Routines and Rituals
3	I must perform something in a particular order (other than routines for bedtime or starting your day)	Routines and Rituals	Routines and Rituals
4	I require going specific routes to familiar destinations	Routines and Rituals	Routines and Rituals
5	I am a “good sport”	Social Flexibility	Social Flexibility
6	I have difficulty with changes in my routine/schedule	Transitions/Change	Transitions/Change
7	I share my possessions	Social Flexibility	Social Flexibility
8	I am interested in other people’s interests and hobbies	Social Flexibility	Social Flexibility
9	**I build on the ideas of others in conversations**	Social Flexibility	Generativity
10	*I closely follow rules*	-	Transitions/Change
11	I am perfectionistic and do not tolerate error or small deviations	Transitions/Change	Transitions/Change
12	I insist on sameness	Transitions/Change	Transitions/Change
13	I repeatedly talk about, write, or draw the same objects	Special Interests	Special Interests
14	*I often pretend to be the same character*	-	Special Interests
15	**I insist on carrying around something with me**	Special Interests	Routines and Rituals
16	My special interests interfere with conversation	Special Interests	Special Interests
17	I become more sociable if discussing my special interests	Special Interests	Special Interests
18	I like to know everything about a topic	Special Interests	Special Interests
19	*I enjoy categorizing information*	-	Special Interests
20	I complain when asked to do things differently	Transitions/Change	Transitions/Change
21	I cannot “shift gears” even if I am told to do so	Transitions/Change	Transitions/Change
22	I have difficulty taking turns	Social Flexibility	Social Flexibility
23	I get upset when losing a game	Social Flexibility	Social Flexibility
24	I can easily generate new ideas and can brainstorm	Generativity	Generativity
25	I think “outside of the box”	Generativity	Generativity
26	I am an independent and creative problem solver	Generativity	Generativity
27	I am generally insistent and like things to stay the way they are	Transitions/Change	Transitions/Change

*Note:* Italicized items indicate the item was removed from FS-SR; bolded items indicate items that loaded on a different factor in the FS-SR (relative to the original FS).
